# Ethanol exposure during the third trimester equivalent does not affect GABA_A_ or AMPA receptor-mediated spontaneous synaptic transmission in rat CA3 pyramidal neurons

**DOI:** 10.1186/s12952-015-0041-9

**Published:** 2015-12-02

**Authors:** Brian Charles Baculis, Carlos Fernando Valenzuela

**Affiliations:** Department of Neurosciences, School of Medicine, University of New Mexico Health Sciences Center, Albuquerque, NM 87131 USA

**Keywords:** Ethanol, Alcohol, Development, Synaptic, Transmission, GABA, Glutamate, Electrophysiology, Channel, Receptor

## Abstract

**Background:**

Ethanol exposure during the rodent equivalent to the 3^rd^ trimester of human pregnancy (i.e., first 1–2 weeks of neonatal life) has been shown to produce structural and functional alterations in the CA3 hippocampal sub-region, which is involved in associative memory. Synaptic plasticity mechanisms dependent on retrograde release of brain-derived neurotrophic factor (BDNF) driven by activation of L-type voltage-gated Ca^2+^ channels (L-VGCCs) are thought to play a role in stabilization of both GABAergic and glutamatergic synapses in CA3 pyramidal neurons. We previously showed that ethanol exposure during the first week of life blocks BDNF/L-VGCC-dependent long-term potentiation of GABA_A_ receptor-mediated synaptic transmission in these neurons. Here, we tested whether this effect is associated with lasting alterations in GABAergic and glutamatergic transmission.

**Methods:**

Rats were exposed to air or ethanol for 3 h/day between postnatal days three and five in vapor inhalation chambers, a paradigm that produces peak serum ethanol levels near 0.3 g/dl. Whole-cell patch-clamp electrophysiological recordings of spontaneous inhibitory and excitatory postsynaptic currents (sIPSCs and sEPSCs, respectively) were obtained from CA3 pyramidal neurons in coronal brain slices prepared at postnatal days 13–17.

**Results:**

Ethanol exposure did not significantly affect the frequency, amplitude, rise-time and half-width of either sIPSCs or sEPSCs.

**Conclusions:**

We show that an ethanol exposure paradigm known to inhibit synaptic plasticity mechanisms that may participate in the stabilization of GABAergic and glutamatergic synapses in CA3 pyramidal neurons does not produce lasting functional alterations in these synapses, suggesting that compensatory mechanisms restored the balance of excitatory and inhibitory synaptic transmission.

## Background

The hippocampus, a brain region located in the medial portion of the temporal lobe, is involved in memory formation, learning, and mood. A number of studies have documented alterations in these processes both in humans and animals exposed to ethanol during development [1]. Deficits in hippocampal function are thought to contribute to these alterations, playing a role the pathophysiology of fetal alcohol spectrum disorders [2, 3]. Although the mechanisms responsible for the hippocampal alterations associated with developmental alcohol exposure are not well understood, several studies suggest that these are, in part, a consequence of damage to neurons located in the CA3 sub-region, which is involved in associative memory [1, 4].

Prenatal exposure to ethanol in rodents (equivalent to the 1^st^ and 2^nd^ trimesters of human pregnancy), has been shown to induce structural and functional changes in the CA3 sub-region. Gestational exposure to ethanol triggered the formation of a hypertrophic infra-pyramidal mossy fiber bundle in the CA3 sub-region [5, 6]. A reduction in ^3^H-vinlidene kainate binding sites was demonstrated in the CA3 *stratum lucidum* of the ventral hippocampus from 45 day-old rat offspring exposed to ethanol during fetal development [7]. Using electron microscopy, Tanaka et al [8] showed that prenatal ethanol exposure decreases the number of synapses in the CA3 sub-region at gestational day 21. Studies suggest that exposure during periods equivalent to the human 3^rd^ trimester of pregnancy can have even more significant effects on this hippocampal sub-region. West and Hamre [9] reported that exposure to ethanol between postnatal day (P) 1 and P10 was associated with the presence of aberrant intra-pyramidal and infra-pyramidal mossy fibers across the CA3 sub-region. Binge-like ethanol exposure during P4-P10 (but not gestational days 1–20) decreased the number and density of pyramidal cells in this sub-region [10, 11]. A similar finding was reported by Miki et al [12] who detected a reduction in CA3 pyramidal neuron number in rats exposed to ethanol between P10 and P15. However, it is noteworthy that studies using both guinea pigs and rats have failed to detect alterations in the number of pyramidal neurons in this hippocampal sub-region [13, 14]. Therefore, several studies have investigated the possibility that developmental ethanol exposure impairs the function of CA3 neurons rather than affecting their morphology.

An electrophysiological study with 50–70 day-old offspring from rats exposed to ethanol throughout gestation reported a reduction in the frequency of high potassium-induced epileptiform bursts in the CA3 *stratum radiatum* [15]. Galindo et al [16] found that acute ethanol exposure increased network-driven giant depolarizing potentials in CA3 pyramidal neurons from neonatal rats, an effect that is likely a consequence of increased GABA_A_ receptor-mediated excitatory synaptic transmission. It was also demonstrated that these immature neurons do not develop tolerance to this effect of ethanol [17]. Acute and repeated ethanol exposure between P2 and P6 was shown to inhibit brain-derived neurotrophic factor (BDNF)- and L-type voltage-gated Ca^2+^ channel (L-VGCC)-dependent long-term potentiation of GABA_A_ receptor-mediated spontaneous postsynaptic currents in CA3 pyramidal neurons [18]. BDNF/L-VGCC-dependent plasticity mechanisms are thought to play a role in the stabilization of both GABAergic and glutamatergic synapses in developing hippocampal neurons [19–21]. Based on these findings, we hypothesized that the ethanol-induced alterations of BDNF/L-VGCC-dependent synaptic plasticity result in a persistent reduction in both GABAergic and glutamatergic synaptic currents in CA3 pyramidal neurons. To test this hypothesis, we exposed neonatal rats to ethanol from P3–P5 and measured GABA_A_ receptor- and AMPA receptor-dependent spontaneous postsynaptic currents at P13–P17 using patch-clamp slice electrophysiological techniques.

## Results

Pups were exposed to high doses of ethanol in vapor chambers between P3-5, as described below. Average pup weights were: P3 (control = 7.7 ± 0.1 g, *n* = 13; ethanol = 7.7 ± 0.2 g, *n* = 13), P5 (control = 10.5 ± 0.3 g, *n* = 14; ethanol = 8.6 ± 0.3 g, *n* = 13), P10 (control = 20.3 ± 0.4 g, *n* = 11; ethanol = 17.6 ± 1.0 g, *n* = 10), P15 (control = 35.3 ± 1.0 g, *n* = 12; ethanol = 30.1 ± 1.5 g, *n* = 11) and P20 (control = 49.9 ± 2.0 g, *n* = 10; ethanol = 43.5 ± 2.3 g, *n* = 12). Two-way ANOVA revealed a significant decrease in weight in the ethanol group at P15 and P20 (age: F (4, 109) = 407.0, *P* <0.0001; ethanol treatment: F (1, 109) = 19.55, *P* <0.0001; Interaction: F (4, 109) = 2.557, *P* = 0.04; *P* = 0.05 by Bonferroni’s test at these ages). In a previous study [22], we did not find a significant effect of this ethanol exposure paradigm on pup body weight, suggesting that offspring from different batches of timed-pregnant Sprague-Dawley rats may display differential sensitivity to ethanol. This may be related to exposure of animals to different stress levels during transport or housing (e.g., exposure to new animal care personnel). The average numbers of pups/litter at the start of the exposure paradigm (P3) were 9.8 ± 0.6 (*n* = 14 litters) and 10.1 ± 0.4 (*n* = 14 litters) for the control and ethanol groups, respectively (U = 97.5; *P* =0.99 by Mann-Whitney test). The average concentration of ethanol in the vapor chamber was 7.9 ± 0.24 g/dl (*n* = 7 rounds of exposure). We have previously shown that this exposure paradigm results in peak serum ethanol levels of 0.33 g/dl (73 mM) in the pup and 0.02 g/dl (5 mM) in the dam [22].

**Fig 1:**
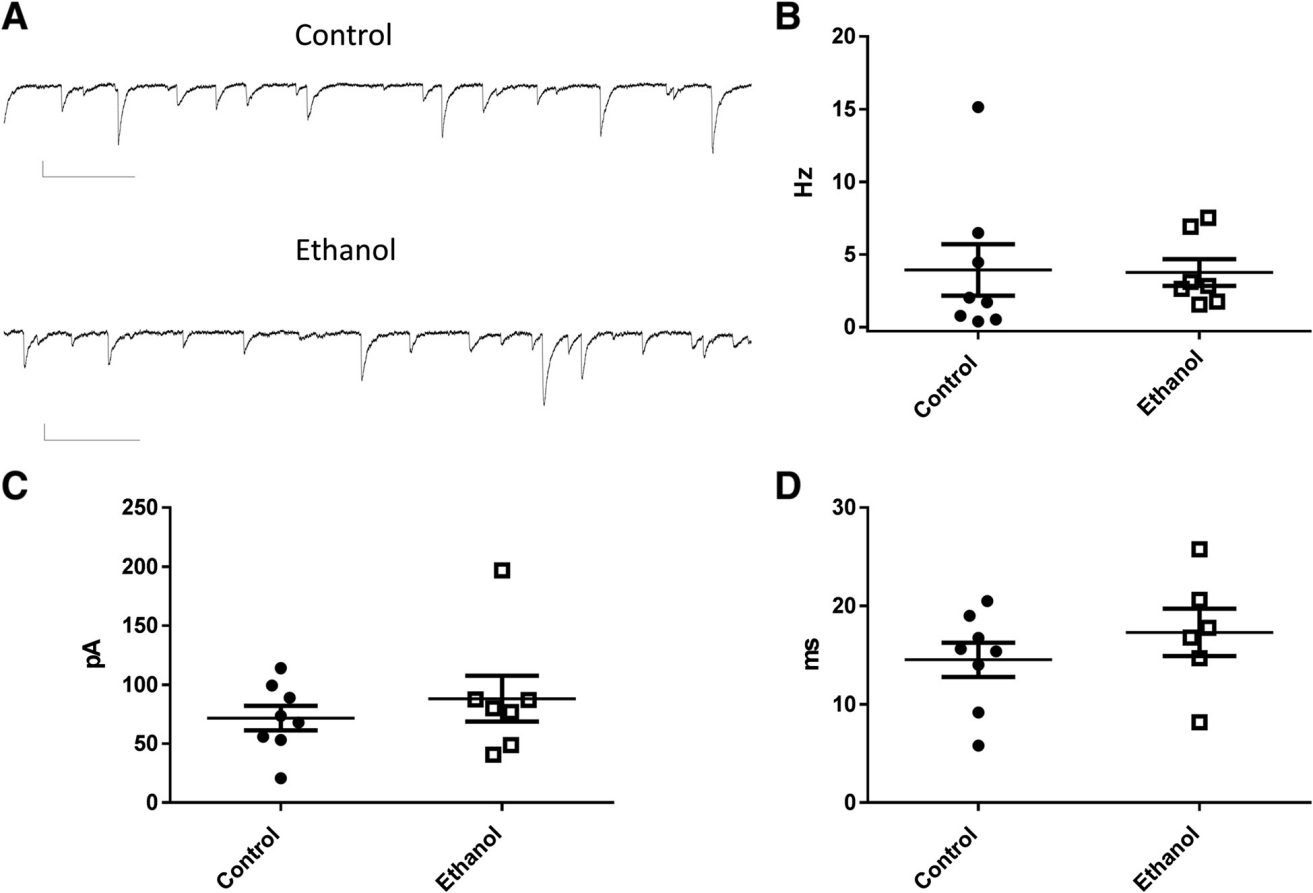
Third trimester-equivalent ethanol exposure did not significantly affect spontaneous inhibitory postsynaptic currents (sIPSCs) in CA3 pyramidal neurons. **a** Sample traces illustrating sIPSC recordings obtained from a postnatal day 15 control rat exposed to air and a postnatal day 16 rat exposed to ethanol. Scale bars = 66.7 pA and 256 ms. Ethanol exposure did not significantly affect sIPSC frequency (**b**), amplitude (**c**) or half-width (**d**)

We evaluated the impact of this ethanol exposure paradigm on GABA_A_ receptor-mediated spontaneous inhibitory postsynaptic currents (sIPSCs). Slice electrophysiological recordings were performed at P13-17. Fig. 1a shows examples of sIPSCs recorded from CA3 pyramidal neurons in slices from control and ethanol treated rats. Neither the frequency of these events (U = 20; *P* = 0.39 by Mann-Whitney test) nor their amplitude (U = 26; *P* = 0.86 by Mann-Whitney test) were significantly different between treatment groups (Fig. 1b–c). Likewise, the half-width of the events was not significantly affected by ethanol exposure (t(12) = 0.96; *P* = 0.35 by t-test; Fig. 1d) nor was the rise time (control = 2.04 ± 0.42 ms, *n* = 8; ethanol = 1.71 ± 0.53 ms, *n* = 6; t(12) = 0.48; *P* = 0.63 by t-test).

**Fig 2:**
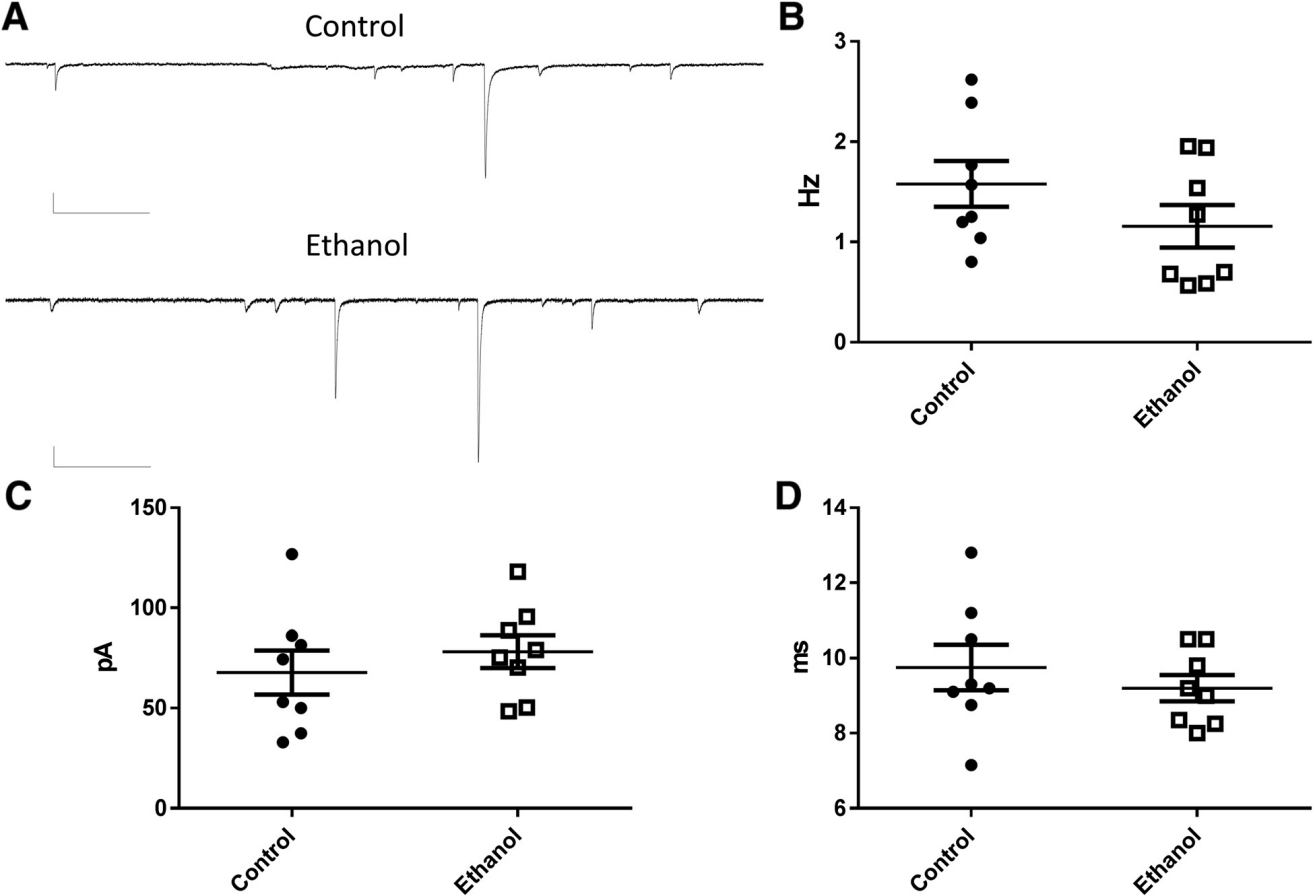
Third trimester-equivalent ethanol exposure did not significantly affect spontaneous excitatory postsynaptic currents (sEPSCs) in CA3 pyramidal neurons. **a** Sample traces illustrating sEPSC recordings obtained from a postnatal day 17 control rat exposed to air and a postnatal day 15 rat exposed to ethanol. Scale bars = 66.7 pA and 328 ms. Ethanol exposure did not significantly affect sEPSC frequency (**b**), amplitude (**c**) or half-width (**d**)

We then assessed the impact of P3-5 ethanol exposure on AMPA receptor mediated sEPSCs. Slice electrophysiological recordings were also performed at P13-17. Sample sEPSC recordings are shown in Fig. 2a. Neither the frequency of these events (t(14) = 1.35; *P* = 0.19 by t-test) nor their amplitude (t(14) = 0.76; *P* = 0.45 by t-test) were significantly different between treatment groups (Fig. 2b-c). Moreover, neither the sEPSC half-width (t(14) = 0.78; *P* = 0.44 by t-test; Fig. 2d) nor the rise time (control = 1.82 ± 0.07 ms, *n* = 8; ethanol = 1.71 ± 0.11 ms, *n* = 8, t(14) = 0.77; *P* = 0.45 by t-test) were significantly affected by ethanol exposure. Spontaneous EPSCs triggered by glutamate release from mossy fibers have been demonstrated to have amplitudes ≥100 pA [23]. Therefore, we analyzed the effect of ethanol exposure on these large events. As shown in Table 1, ethanol exposure did not significantly affect the frequency, amplitude, rise-time or half-width of these events. Small events with an amplitude ≤100 pA were not significantly affected either. The percent of events with amplitudes ≥100 pA was 17.5 ± 4.6 % for the control group and 17.2 ± 2.5 % for the ethanol group (t(14) = 0.063; *P* = 0.95 by unpaired t-test; *n* = 8).

**Table 1:** Summary of characteristics of large (≥100 pA) and small (≤ 100 pA) sEPSCs

Parameter	Control	Ethanol	Statistics
Frequency (Hz)			
Large events	0.35 ± 0.04 (*n* = 6)	0.24 ± 0.05 (*n* = 6)	U = 12; *P* = 0.14
Small events	1.35 ± 0.25 (*n* = 8)	0.96 ± 0.16 (*n* = 8)	t(14) = 1.26; *P* = 0.22
Amplitude (pA)			
Large events	232.2 ± 14 (*n* = 8)	255.2 ± 26 (*n* = 8)	t(14) = 0.78;*P* = 0.44
Small events	34.6 ± 1.9 (*n* = 8)	41.5 ± 3.1 (*n* = 8)	t(14) = 1.88; *P* = 0.08
Rise time (ms)			
Large events	2.08 ± 0.11 (*n* = 8)	1.86 ± 0.10 (*n* = 7)	t(13) = 1.33; *P* = 0.2
Small events	1.5 ± 0.12 (*n* = 8)	1.47 ± 0.13 (*n* = 8)	t(14) = 0.16; *P* = 0.86
Half-width (ms)			
Large events	10.08 ± 0.67 (*n* = 8)	8.9 ± 0.46 (*n* = 7)	t(13) = 1.38; *P* = 0.18
Small events	8.76 ± 0.69 (*n* = 8)	8.3 ± 0.86 (*n* = 8)	t(14) = 0.4; *P* = 0.69

## Discussion

Our ethanol exposure paradigm did not significantly affect the properties of sIPSCs, a finding that does not support the hypothesis that ethanol exposure during this critical period, when CA3 pyramidal neurons express BDNF/L-VGCC-dependent synaptic plasticity, produces a lasting impairment in GABA_A_ receptor-dependent synaptic transmission. We did not observe changes in sIPSC frequency, amplitude, rise-time or half-width, indicating that ethanol exposure neither affects spontaneous GABA release nor the function of postsynaptic GABA_A_ receptors. These findings suggest that ethanol did not have an overall effect on the function of GABAergic interneurons that provide inhibitory inputs to the soma and proximal dendrites of CA3 pyramidal neurons. Due to space-clamp limitations, GABAergic inputs to distal dendritic sites cannot be reliably measured using somatic patch-clamp electrodes [24]. Therefore, we cannot discount the possibility that these were affected by ethanol exposure. This issue could be addressed in the future using current-clamp recordings of spontaneous inhibitory postsynaptic potentials or voltage-clamp experiments of IPSCs evoked by electrical stimulation of distal inputs. As mentioned above, BDNF/L-VGCC-dependent long-term potentiation of GABAergic transmission is thought to play a role in stabilizing GABA_A_ receptor-containing synapses in the soma and/or proximal dendrites of CA3 pyramidal neurons [19]. Therefore, it is somewhat surprising that ethanol-induced inhibition of this plasticity mechanism did not affect the properties of GABA_A_-dependent sIPSCs [18]. A potential explanation for the lack of a lasting effect is that the action of ethanol is transient, occurring only while this agent is present during the 3 h/day exposure on P3-P5 (and several hours/day post-exposure while ethanol is being metabolized) and reversing at some point between this period and the time at which the electrophysiological recordings were acquired (P13-P17). We previously demonstrated that ethanol vapor exposure from P2-P16 causes a significantly delay in the acquisition of action potential-independent GABA_A_ receptor mediated postsynaptic currents in CA3 pyramidal neurons [25], suggesting that repeated ethanol exposure encompassing a longer period of development could alter plasticity mechanisms involved in the maturation of GABAergic synapses.

Out studies also failed to demonstrate an effect of ethanol exposure on the frequency, amplitude, rise-time or half-width of AMPA receptor-mediated sEPSCs, including large events likely mediated by glutamate release from mossy fibers. These results suggest that ethanol did not have lasting effects on glutamatergic transmission at the pre- or post-synaptic levels. The lack of an effect of ethanol on large sEPSCs is unexpected given that BDNF/L-VGCC-dependent synaptic plasticity has been shown to strengthen mossy fiber-to-CA3 pyramidal neuron synapses during postnatal development [20]. Moreover, we have previously demonstrated that acute ethanol exposure inhibits glutamate release and AMPA receptor-mediated EPSCs in slices from P3-P10 rats [26] and it would have been expected for these acute effects to cause lasting compensatory pre- and/or post-synaptic alterations in glutamatergic transmission [27–29]. As was the case for GABAergic transmission, it is possible that neurons were able to counteract the effects of ethanol, leading to a normalization of glutamatergic transmission at the time of our electrophysiological experiments. For instance, neurons could have engaged alternative pathways involved in synapse stabilization, including upregulation of modulatory neurotransmitter systems (e.g., serotonin) [30, 31].

## Conclusions

We demonstrate here that a 3^rd^ trimester-equivalent ethanol exposure paradigm that significantly impairs synaptic plasticity mechanisms thought to be involved in the stabilization of GABAergic and glutamatergic synapses in CA3 pyramidal neurons does not produce lasting alterations in spontaneous synaptic transmission mediated by GABA_A_ and AMPA receptors. These findings suggest that neonatal CA3 pyramidal neurons deploy compensatory mechanisms in response to this exposure paradigm that ultimately restore the balance of excitatory and inhibitory synaptic transmission. It should be emphasized, however, that even if the effects of our P3–P5 exposure paradigm are transient, these could still alter the trajectory of developmental processes other than the refinement of GABAergic and glutamatergic synaptic transmission [32] and this issue should be investigated in the future.

## Methods

All chemicals were obtained from Sigma-Aldrich (St. Louis, MO), except when noted. The University of New Mexico Health Sciences Center Institutional Animal Care and Use Committee approved the procedures followed in this study. Timed-pregnant Sprague-Dawley rats (Harlan, Indianapolis, IN) were used in these experiments. The gestational age upon arrival to our animal facility was 12–15 days. Between P3 and P5, pups were exposed to air (control) or ethanol for 3 h/day using a previously described vapor inhalation chamber paradigm [22]. We previously showed that the paradigm minimally disrupts maternal care [22]. Coronal brain slices (300 μm) containing the dorsal hippocampal formation were prepared from ketamine-anesthetized rats at P13–17 using a vibrating slicer (Leica Microsystems, Bannockburn, IL) and sucrose-based cutting solution, as previously described [22]. Subsequently, slices were allowed to recover in oxygenated artificial cerebral spinal fluid containing (in mM): NaCl, 125; KCl, 2; NaH_2_PO_4_, 1.3; NaCO_3_, 26; glucose, 10, CaCl_2_, 2; MgSO_4_, 1 at 35 °C for 40 min in a water bath. The chamber containing the slices was then taken out of the water bath and stored at 21–22 °C for at least 30 min prior to the start of electrophysiological recordings. Neurons were visualized using an Olympus BX50WI upright microscope (Olympus, Center Valley, PA) equipped with infra-red Differential Interference Contrast optics and connected to a charge-coupled device camera (CCD100, DAGE-MTI, Michigan City, IN). Whole-cell patch-clamp electrophysiological recordings were obtained at -70 mV and 32 °C using an Axopatch 200B amplifier (Molecular Devices, Sunnyvale, CA). Spontaneous IPSCs were recorded in presence of 3 mM kynurenate using an internal solution containing (in mM): KCl, 135; HEPES, 10; EGTA, 0.5; MgCl_2_, 2; ATP-Mg, 5; GTP-Na, 1; QX-314-Cl, 1; pH 7.3 with KOH. These events were blocked by the GABA_A_ receptor antagonist, gabazine (Tocris, Bristol, U.K.) (50 μM; *n* = 3). AMPA receptor-mediated sEPSCs were recorded in presence of 50 μM gabazine using an internal solution containing (in mM): K-Gluconate, 120; KCl, 15; EGTA, 0.1; HEPES, 10; MgCl_2_, 4; ATP-Mg, 4; GTP-Na, 0.3; phosphocreatine, 7; pH 7.3 with KOH. These events were blocked by the AMPA receptor antagonist, 2,3-Dioxo-6-nitro-1,2,3,4-tetrahydrobenzo[f]quinoxaline-7-sulfonamide (NBQX, 10 μM; *n* = 3). The access resistance was between 5 and 26 MΩ and if it changed more than 30 %, the recording was rejected. Mini-Analysis program (Synaptosoft, Decatur, GA) was used to analyze the electrophysiological data. Statistical analyses were performed with Prism Version 6.05 (GraphPad Software, San Diego, CA). Data were initially analyzed with the Kolmogorov-Smirnov normality test and if they followed a normal distribution, they were subsequently analyzed using parametric tests (t-tests or two-way ANOVA). If data did not pass the normality test, they were analyzed using two-tailed Mann-Whitney tests. Data are presented as mean ± SEM. The unit of determination is the average of results obtained with slices from a single animal (1–3 cells/animal; each cell from a different slice).

## Abbreviations

AMPA: α-amino-3-hydroxy-5-methyl-4-isoxazolepropionic acid
ANOVA: analysis of variance
BDNF: brain-derived neurotrophic factor
EGTA: ethylene glycol tetra acetic acid
GABA_A_: γ-amino butyric acid receptor, type-A
HEPES: 4-(2-hydroxyethyl)-1-piperazineethanesulfonic acid
L-VGCC: L-type voltage-gated Ca^2+^ channel
NBQX: 2,3-Dioxo-6-nitro-1,2,3,4-tetrahydrobenzo[f]quinoxaline-7-sulfonamide
P: postnatal day
QX-314: N-(2,6-Dimethylphenylcarbamoylmethyl) triethylammonium chloride
sEPSC: spontaneous excitatory postsynaptic current
sIPSC: spontaneous inhibitory postsynaptic current
